# Protective effect of *Cordyceps sinensis* extract on lipopolysaccharide-induced acute lung injury in mice

**DOI:** 10.1042/BSR20190789

**Published:** 2019-06-25

**Authors:** Shuiqiao Fu, Weina Lu, Wenqiao Yu, Jun Hu

**Affiliations:** 1 Department of Surgery Intensive Care Unit, The First Affiliated Hospital Zhejiang University School of Medicine, Hangzhou 310003, Zhejiang, China; 2 Department of Surgery Intensive Care Unit, The Second Affiliated Hospital Zhejiang University School of Medicine, Hangzhou 310009, Zhejiang, China

**Keywords:** acute lung injury, Cordyceps sinensis, lipopolysaccharide, COX-2, NF-κB p65, iNOS

## Abstract

**Background:** To study the protective effect of *Cordyceps sinensis* extract (Dong Chong Xia Cao in Chinese [DCXC]) on experimental acute lung injury (ALI) mice.

**Methods and results:** ALI model was induced by intratracheal-instilled lipopolysaccharide (LPS, 2.4 mg/kg) in BALB/c male mice. The mice were administrated DCXC (ig, 10, 30, 60 mg/kg) in 4 and 8 h after receiving LPS. Histopathological section, wet/dry lung weight ratio and myeloperoxidase activity were detected. Bronchoalveolar lavage fluid (BALF) was collected for cell count, the levels of tumor necrosis factor-α (TNF-α), interleukin-1β (IL-1β), interleukin-6 (IL-6) and nitric oxide (NO) in BALF was detected by ELISA, the protein and mRNA expression of nuclear factor-κB p65 (NF-κB p65), inducible NO synthase (iNOS) and cyclooxygenase-2 (COX-2) in lung tissue was detected by Western blot and RT-PCR. The result showed that DCXC could reduce the degree of histopathological injury, wet/dry weight ratio (W/D ratio) and myeloperoxidase activity (*P*<0.05) with a dose-dependent manner. The increased number of total cells, neutrophils and macrophages in BALF were significantly inhibited by DCXC treatment (*P*<0.05). The increased levels of TNF-α, IL-1β, IL-6 and NO in BALF after LPS administration was significantly reduced by DCXC (*P*<0.05). In addition, the increased protein and mRNA levels of iNOS, COX-2 and NF-κB p65 DNA binding ability in LPS group were dose-dependently reduced by DCXC treatment (*P*<0.05).

**Conclusion:** DCXC could play an anti-inflammatory and antioxidant effect on LPS-induced ALI through inhibiting NF-κB p65 phosphorylation, and the expression of COX-2 and iNOS in lung. The result showed that DCXC has a potential protective effect on the ALI.

## Introduction

Acute lung injury (ALI) and its severest stage acute respiratory distress syndrome (ARDS) will finally lead to a multiple organ dysfunction syndromes (MODS) in lung tissue [1]. MODS characterized with a pulmonary infiltrate, hypoxemia, and the absence of an elevated pulmonary capillary wedge pressure. ALI has a high mortality rate, and it is mainly caused by sepsis, burns and severe trauma, and systemic inflammatory reaction based on diffuse lung cell injury (DPI) [2]. Lung is one of the most easily invaded organs and also the first target organs in endotoxemia. ALI is a common acute and critical disease in clinic. It has a complicated pathogenesis, the pathological characterizations of ALI are pulmonary edema and atelectasis, which will lead to an inflammation and increased permeability of lung tissue, and this increased permeability will finally lead to a gas exchange dysfunction [3,4]. The specific manifestations of ALI are the increase of pro-inflammatory cytokines such as tumor necrosis factor-α (TNF-α), interleukin-6 (IL-6) and interleukin-1β (IL-1β), as well as the release of other mediators caused by cascade amplification, resulting in increased pulmonary capillary permeability, interstitial edema, neutrophil exudation and a series of inflammation [5,6].

Pulmonary macrophages (PAM) are the main inflammatory cells in alveoli in the early stage of ALI, which are widely distributed in alveoli and airway epithelial cells [7,8]. PAM is the first line of defense against pathogenic microorganisms and lung injury. Under the influence of lipopolysaccharide (LPS) and other factors, PAM releases a large number of pro-inflammatory mediators, especially TNF-α. Over-increased inflammatory factors will eventually lead to uncontrolled inflammation, leading to body damage, ALI and ARDS, and in severe cases, it will promote multiple organ failure (MOF) [9,10]. Since the complex etiology and high mortality of ARDS, it has been a hot topic in clinical research. Although some progress has been made in the diagnosis and treatment of ALI/ARDS in recent years, there is still a high mortality rate [11,12], the mortality rate of patients with ALI is 38.5%, and the mortality rate of that of patients with ARDS is 41.1%. Therefore, it is urgent and necessary to find more effective drugs to control the symptoms and ultimately to treat ALI/ARDS.

As we all know, China is the birthplace of traditional Chinese medicines (TCM), TCM is rich in species and low in cost [13]. Many studies show that TCM has a good anti-inflammatory effect and enhance the immunity of the body, which can play a certain role in the prevention and protection of acute organ injury including improvement of symptoms, quantity of life, and lung function [14,15]. *Cordyceps* sinensis, or Dong Chong Xia Cao in Chinese (DCXC), is a special type of medicinal mushroom, which forms on an insect larva infected by the *C.* sinensis fungus [16]. DCXC rich in many bioactive constitutes, such as polysaccharide, nucleoside, amino acid, polypeptide, protein and sterol [16]. Recent research on DCXC mainly focused on chemical components and the related pharmacological functions. Some researchers have reported that DCXC could decrease oxidative stress in human lung epithelial cells. While modern pharmacological researches reported that DCXC has many functions as antitumor, anti-inflammatory, anti-aging, antioxidant, anti-apoptosis, and can regulate endocrine, respiratory, immune, and nervous systems [17–19]. However, the specific inhibitory mechanisms of DCXC are not clear.

Based on the above, DCXC has a wide pharmacological function, but until now, to the best of our knowledge, there have not any researches for the effect of DCXC on LPS-induced ALI. Hence, here we aimed to investigate the protective effect and clarify the potential action mechanism of DCXC on LPS-induced ALI in mice, which could provide the theoretical basis for the clinical application of DCXC in ALI.

## Methods and materials

### Materials and reagents

Mouse tumor necrosis factor-α (TNF-α, Batch No 978941030), interleukin-1 β (IL-1β, Batch No 9711891103), interleukin-6 (IL-6, Batch No 1321213119), nitric oxide (NO, Batch No 13213141033) and mouse myeloperoxidase (MPO, Batch No 132111031103) were purchased from Wuhan Boster Biology Technology co. ltd. (Wuhan, China). Mouse anti human nuclear factor-κB p65 (NF-κB p65), phosphor NF-κB p65 (pNF-κB p65), cyclooxygenase-2 (COX-2), inducible NO synthase (iNOS) and β-actin antibody were purchased from Santa Cruz (CA, U.S.A.). NF-κB p65 transcription factor assay kit was purchased from Abnova Corporation (Taiwan). Enhanced chemiluminescence detection kit obtained from Merck-Millipore (Darmstadt, German). LPS purchased from Sigma–Aldrich (Darmstadt, German).

*Cordyceps sinensis* extract (batch number: 030522), a light brown dry powder, containing the components of 0.053% cordycepin (3-deoxyadenosine), 4–7% cordycepin acid (D-mannitol) and 0.078% adenosine, was obtained from Shenyang Zhongtian Bioengineering Co., Ltd.

### Animals

Sixty adult male BALB/c mice (body weight 20 ± 2 g) aged 8 weeks were provided by the animal experimental center of the Zhejiang University (Hangzhou, China). Six mice were raised in one polyacrylic cage, and all the mice were quarantined for 1 week before the use. All the mice were reared in the animal experimental center of Zhejiang University in SPF grade environment with free access to food and water (24 ± 1°C, 50 ± 5% of humidity and 12-h day/night cycle). The mice were received humane care in the terms of National Institutes of Health Guidelines of the U.S.A. (National Research Council of U.S.A., 1996) and the University ethical regulations of Zhejiang University.

### Experimental design

All animal study protocols were approved by the animal care and use committee of Zhejiang University. Sixty adult male BALB/c mice aged 8 weeks were randomly divided into five groups: the vehicle group, LPS group, DCXC 10 mg/kg + LPS group, DCXC 30 mg/kg + LPS group and DCXC 60 mg/kg + LPS group. The mice were intratracheal instillation of 2.4 mg/kg of LPS in saline (or with saline as a control) under anesthesia using sodium pentobarbital (40 mg/kg). The mice were given DCXC (ig, 10, 30, 60 mg/kg) or saline (vehicle and model group) in 4 and 8 h after receiving LPS, respectively. After 12 h of LPS stimulation, mice were executed by intraperitoneal injection of excessive sodium pentobarbital (80 mg/kg). The right lower lobe lung tissue of each group was fixed in 10% formalin.

### Histopathological analysis

Lower lobe of right lung tissue in each group was fixed in the 40 g/l formaldehyde solution overnight. The fixed lung tissues were embedded in paraffin, cut into 5-μm thickness sections and then stained with hematoxylin and eosin (H&E) in terms of the routine histopathological examination. The final stained sections were photographed under a light microscope (BX-50 Olympus) at 200× magnification.

### Detection of wet/dry weight ratio and lung index for lung tissue

After 12 h of LPS stimulation, the mice were killed by anesthesia. The right upper lobe lung tissues of each group were harvested, the water on the surface of lung tissues was dried with filter paper. The lung weight of each group was weighed by precise electronic balance, namely wet weight. Pulmonary tissues were numbered and placed in an oven at 80°C for 48 h until the weight of lung tissues did not change. The weight of lung tissues in each group was weighed again, that is, dry weight [20]. The ratio of wet weight/dry weight of lung tissue in mice was calculated as W/D ratio to evaluate the degree of pulmonary edema. In addition, the body weight of mice in each group were weighted, and the lung index (lung index % = wet lung weight/body weight × 10^3^) was calculated to further confirm the degree of pulmonary edema.

### Collection of bronchoalveolar lavage fluid and cell count

At the end of the experiment (after the stimulation time), the mice in each group were killed by intraperitoneal injection of excessive sodium pentobarbital (80 mg/kg). The mice were fixed on the operating table, disinfected by alcohol, made an incision from the middle of the neck, separated the skin and muscles of the neck, fully exposed the trachea, inserted 18G trocar and fixed with silk thread. Open the chest of mice, cut the sternum, expose lung tissue, and ligate the right lung with silk thread. The left lung was lavage with PBS at 4°C, 0.6 ml each time and then slowly withdrawn after 30 s. The lavage was repeated three times. The liquid extraction rate is more than 90%. Transfer the lavage solution to the centrifugal tube and place it on the ice. Centrifuge it at 4°C and 700 ***g*** for 5 min. The supernatant was separated into sterilized EP tubes, then placed in a low-temperature refrigerator at −80°C for testing. The precipitated cells were suspended with 50 μl PBS, smeared, then stained with Diff-Quik stain. The total cells, neutrophils and macrophages in bronchoalveolar lavage fluids (BALFs) of mice in each group were counted by cell counting plate under optical microscope (300 cells per smear).

### Detection of TNF-α, IL-6, IL-1β and NO expression in BALF of mice by ELISA

The expressions of TNF-α, IL-6, IL-1β and NO in BALF of mice in each group were detected by ELISA kit instructions (Wuhan Boster Biology Technology co. ltd., Wuhan, China).

### Determination of myeloperoxidase activity in lung tissue of mice

Preparation of lung tissue homogenate: pulmonary tissue homogenate of mice was harvested, filtered paper was used to absorb dirty blood and exudate, rinsed in cold physiological saline, filtered paper was wiped dry, and appropriate weight of lung tissue was weighed. A total of 5% of pulmonary tissue homogenate was prepared according to the kit protocol from Wuhan Boster Biology Technology co. ltd. (Wuhan, China).

### Western blot assay

The protein expression of phospho NF-κB p65, COX-2 and iNOS in lung tissue of each group were detected by Western blot. First, use the pre-cold PBS buffer to wash the frozen lung tissues. Take proper amount of lung tissue into the pre-cold tissue homogenizer. Add proper volume of homogenate buffer (50 mmol/l, pH 7.5 Tis-HCl, 150 mmol/l NaCl, 1 mmol/l phenyl methyl sulfonyl fluoride, 1 mg/ml aprotinin, 4 mg/ml leupeptin), homogenized in ice bath, centrifugation at 4°C, 10000 rpm/min, collect the supernatant, then measured the protein concentration was by BCA method. The samples loaded at 10% SDS/PAGE electrophoresis gel (80 mg protein/hole) for running. PVDF membrane were used for transferring, after antibody incubation, the membrane was imaged at Syngene GBO X gel scan imager after ECL reagent visualization. The relative quantification of protein levels was calculated by image J software.

### NF-κB p65 DNA bonding affinity testing

The NF-κB p65 transcription factor assay kit is a new, non-radioactive and sensitive kit for detecting the binding capacity of NF-κB p65 DNA in nuclear proteins. The kit will pre-fix the dsDNA sequence specifically bound to the bottom of the 96-well plate, and detect the amount of NF-κB p65 bound to dsDNA by antibody–antigen specific binding. The OD values obtained at the wavelength of 450 nm. The binding ability of NF-κB p65 DNA in lung tissue of mice in each group detected according to the instruction of the transcription factor assay kit for NF-κB p65.

### Quantitative RT PCR

A total RNA sample of each lung tissue was extract using Trizol reagent (15596026, invitrogen, Shanghai, China), according to the manufacturer’s introduction. Take 1 μg of total RNA to reverse transcribed cDNA using the Primer-Script TM one step RT-PCR reagent kit (Takara, Shiga, Japan), according to the manufacturer’s protocol. The relative quantification of mRNA expression levels were measured using the SYBR Green I real time PCR kit (CoWin Bioscience Co., Beijing, China). The sequences of all the primer were COX-2 (296 bp): forward 5′-TCAAAAGAAGTGCTGGAAAAGGTT-3′, reverse 5′-TCTACCTGAGTGTCTTT GACTGTG-3′; iNOS (346 bp): forward 5′-ATTCAGATCCCGAAACGC-3′, reverse 5′-CCAGAACCTCCAGGCACA-3′; β-actin (214 bp) forward 5′-CCCACTCCTAAG AGGAGGATG-3′, reverse 5′-AGGGAGACCAAAGCCTTCAT-3′. β-actin was used as an endogenous control for normalize the mRNA expression levels. The changes of the mRNA expression in all the groups were calculated by the method of 2^−ΔΔ*C*^_t_ [21].

### Statistical analysis

Values were represented as mean ± S.D. All statistical comparisons were calculated by means of a one-way ANOVA test followed by Dunett’s *t*-test with SPSS19.0 statistical software. *P*<0.05 and <0.01 were regarded as statistically significant.

## Results

### DCXC improves lung histopathology in LPS-induced ALI

**Figure 1:**
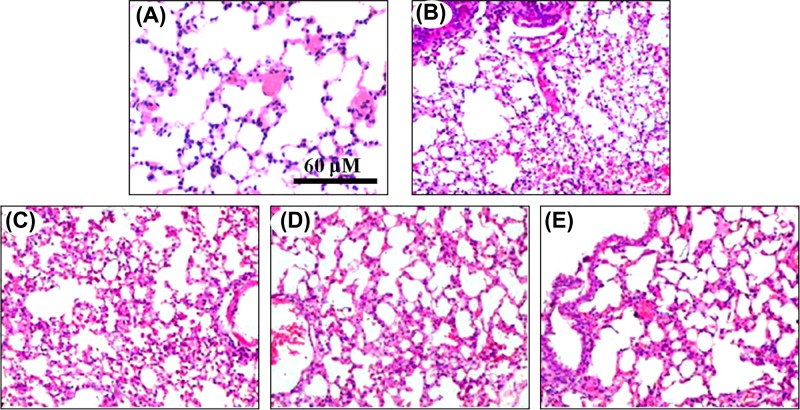
DCXC improves lung histopathology in LPS-induced acute lung injury mice (H&E, scale bar 60 μm, 200×) (**A**) vehicle group, (**B**) LPS model group, (**C**) DCXC 10 mg/kg, (**D**) DCXC 30 mg/kg, (**E**) DCXC 60 mg/kg.

Previous studies have shown that the pathological changes of lung tissue can reflect the ALI induced by LPS [22]. In this experiment, 12 h after LPS-induced ALI in mice, the structure of lung lobules in vehicle group was intact, alveolar wall was not abnormal, no inflammatory cell infiltration, no alveolar cavity exudation, no transparent membrane formation (Figure 1A). In LPS group, a large number of inflammatory cells infiltrated the lung tissue, the alveolar septum became thicker, transparent membrane formed, pulmonary interstitial edema, alveolar cavity exuded, part of the alveolar structure was destroyed and bleeding focus was found (Figure 1B). The interventions of DCXC at doses of 10, 30 and 60 mg/kg could alleviate the pathological damage of lung tissue in LPS mice, and showed dose-dependent manner (Figure 1C-E).

### DCXC can reduce the wet/dry weight ratio and lung index of lung tissue in LPS-induced ALI in mice

**Figure 2:**
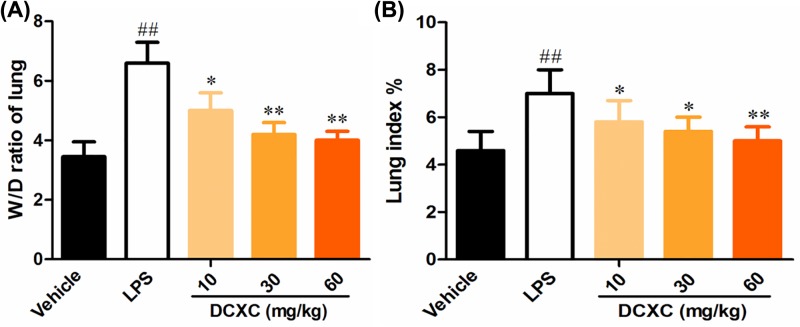
DCXC can reduce the wet/dry weight ratio and lung index of lung tissue in LPS-induced acute lung injury model mice (*n*=12) Wet/dry weight ratio (W/D ratio) (**A**), lung index (wet lung weight/body weight × 10^3^) (**B**). ^##^*P*<0.01 vs vehicle group, *P<0.05, **P<0.01 vs LPS group.

After LPS-induced ALI in mice for 12 h, the wet/dry weight ratio (W/D ratio) of lung tissue responded to the degree of non-cardiogenic pulmonary edema [23]. Compared with the vehicle group, the ratio of W/D ratio and the lung index in LPS group increased significantly (Figure 2, *P*<0.01). Compared with LPS group, the ratio of W/D of lung tissue and the lung index in DCXC (10, 20 and 30 mg/kg) group were significantly lower than that in LPS group (*P*<0.05, *P*<0.01) (Figure 2). These results suggest that LPS can successfully induce ALI model, and DCXC in the doses of 10, 30 and 60 mg/kg can reduce the degree of pulmonary edema in LPS-induced ALI with a dose-dependent manner.

### DCXC can reduce the number of total cells, neutrophils and macrophages in BALF of ALI mice induced by LPS

In Table 1, the number of total cells, neutrophils and macrophages in BALF of LPS group mice were increased significantly, compared with the vehicle group (*P*<0.01). While compared with LPS group, the number of total cells, neutrophils and macrophages in BALF of mice in DCXC (10 mg/kg) group decreased with no significant difference (*P*>0.05); the number of total cells, neutrophils and macrophages in DCXC (20 and 30 mg/kg) group decreased significantly (*P*<0.05, *P*<0.01). These results suggest that DCXC can reduce the number of total cells, neutrophils and macrophages in BALF of LPS-induced ALI mice in a dose-dependent manner (Table 1).

**Table 1:** Number of total cells, neutrophils and macrophages in BALF of mice in each group

Group	Total cells (×10^6^)	Neutrophils (×10^6^)	Macrophages (×10^6^)
Vehicle	0.066 ± 0.013	0.002 ± 0.001	0.060 ± 0.003
LPS	1.470 ± 0.235^##^	0.947 ± 0.200^##^	0.418 ± 0.100^##^
DCXC 10 mg/kg	1.321 ± 0.220	0.900 ± 0.110	0.400 ± 0.053
DCXC 30 mg/kg	1.014 ± 0.210*	0.671 ± 0.091*	0.211 ± 0.132*
DCXC 60 mg/kg	0.340 ± 0.042**	0.208 ± 0.102**	0.163 ± 0.104**

Data are expressed as mean ± S.D. for each group. ^##^*P*<0.01 vs Vehicle group, *P<0.05, **P<0.01 vs LPS group.

### DCXC can reduce the expression of TNF-α, IL-6 and IL-1β in BALF of LPS-induced ALI mice

**Figure 3:**
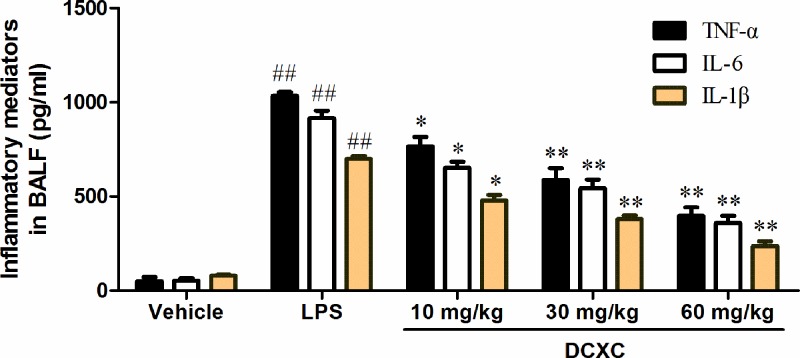
DCXC can reduce the expression of TNF-α, IL-6 and IL-1β in BALF of LPS-induced acute lung injury mice (*n*=12) ^##^*P*<0.01 vs vehicle group, *P<0.05, **P<0.01 vs LPS group.

ELISA results showed that the expression of TNF-α, IL-6 and 1L-1β in LPS group was significantly higher than that in vehicle group (Figure 3, *P*<0.01), while DCXC (10, 30 and 30 mg/kg) could significantly reduce the expression of TNF-α, IL-6 and 1L-1β in BALF compared with LPS group (*P*<0.05, *P*<0.01). The results of DCXC (10, 30 and 30 mg/kg) groups showed that the expression of TNF-α, IL-6 and 1L-1β in BALF decreased gradually with the increase of DCXC intervention dose (Figure 3). DCXC can reduce the expression of TNF-α, IL-6 and 1L-1β in BALF of LPS-induced ALI mice, and there is a dose-dependent relationship.

### DCXC can reduce NO expression in BALF of LPS-induced ALI in mice

**Figure 4:**
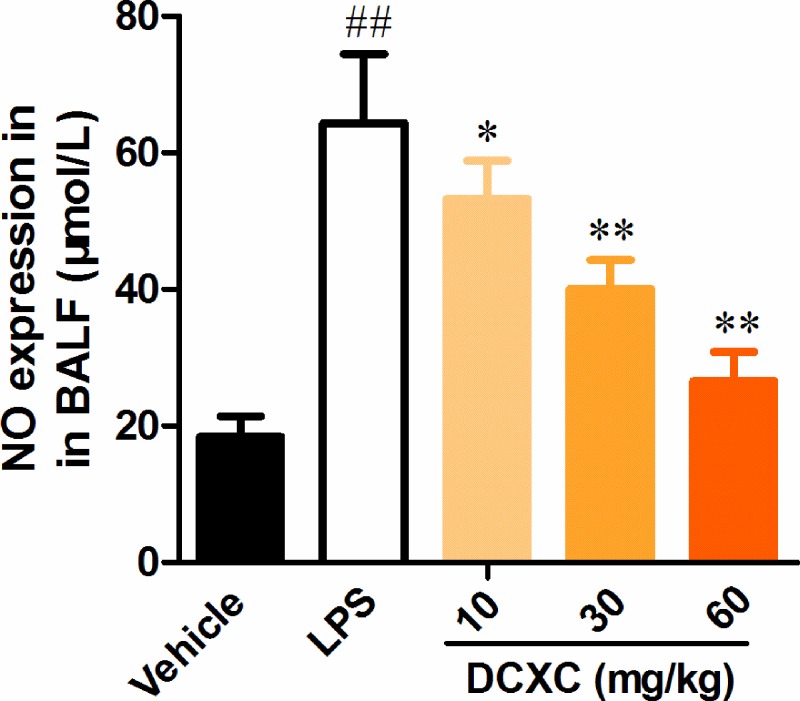
DCXC can reduce NO expression in BALF of LPS-induced acute lung injury mice (*n*=12) ^##^*P*<0.01 vs vehicle group, *P<0.05, **P<0.01 vs LPS group.

ELISA analysis showed that the expression of NO in LPS group was significantly higher than that in vehicle group (Figure 4, *P*<0.05). Compared with LPS group, DCXC could significantly reduce the expression of NO in BALF (*P*<0.05, *P*<0.01). With the increase of DCXC intervention dose, the expression of NO in BALF decreased gradually (Figure 4). DCXC can reduce the expression of NO in BALF of LPS-induced ALI mice with a dose-dependent manner.

### DCXC can inhibit MPO activity in lung tissue of LPS-induced ALI mice

**Figure 5:**
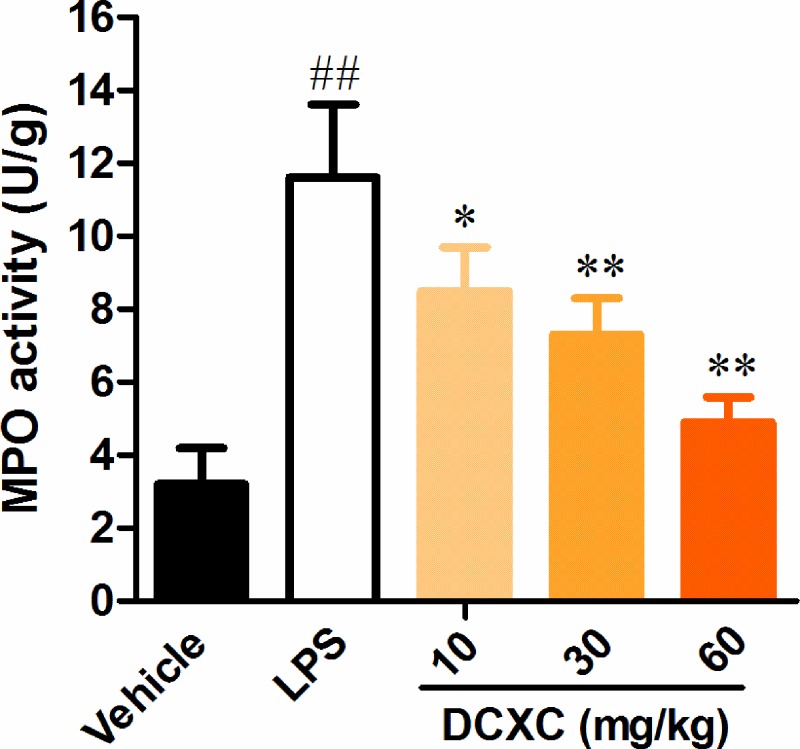
DCXC can inhibit MPO activity in lung tissue of LPS-induced acute lung injury model mice (*n*=12) ^##^*P*<0.01 vs vehicle group, *P<0.05, **P<0.01 vs LPS group.

MPO mainly synthesized and released by neutrophils. The activity of MPO in lung tissue is an important indicator of neutrophil infiltration in inflammatory process [23]. Compared with the vehicle group, the activity of MPO in lung tissue of LPS group increased significantly (Figure 5, *P*<0.01), while the activity of MPO in lung tissue of DCXC (10, 20 and 30 mg/kg) group decreased significantly (*P*<0.05, *P*<0.01) with the increase of DCXC intervention dose (Figure 5). DCXC can inhibit the activity of MPO in lung tissue of LPS-induced ALI mice in a dose-dependent manner.

### DCXC can inhibit the protein expression of phosphorylation NF-κB p65, COX-2 and iNOS in lung tissue of LPS-induced ALI mice

**Figure 6:**
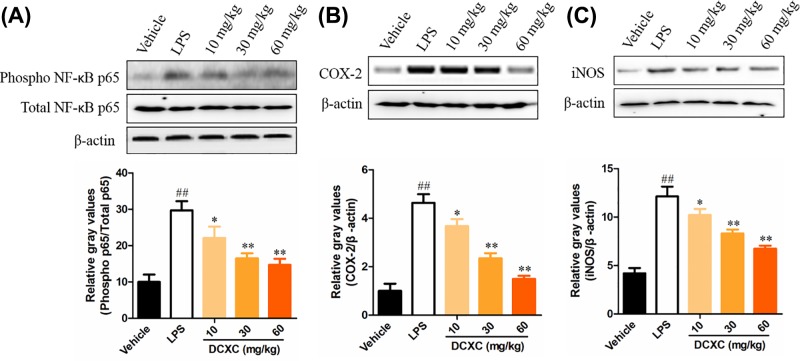
DCXC can inhibit the protein expression of phosphorylated NF-κB p65, COX-2 and iNOS in lung tissue of LPS-induced acute lung injury mice (*n*=12) ^##^*P*<0.01 vs vehicle group, ^*^P<0.05, ^**^P<0.01 vs LPS group

NF-κB signaling pathway plays an important role in the regulation of LPS-induced acute inflammation and injury [24,25]. After 12 h of LPS-induced ALI mice, the protein expression ratio of phosphor NF-κB p65/total NF-κB p65 in lung tissue of LPS group was significantly higher than that of vehicle group (Figure 6A, *P*<0.01). While the protein expression ratio of DCXC (10, 20 and 30 mg/kg) was significantly lower than that of LPS group (Figure 6A, *P*<0.05, *P*<0.01). In addition, we also detected the phosphor IκBα levels after DCXC treatment. However, there are no any changes in DCXC group, compared with vehicle group, the data we did not represent. This result reminds us the potential target of DCXC in ALI mice model.

COX-2 is an important inflammatory mediator downstream of the NF-κB pathway, which plays an important role in the pathogenesis of ALI and is related to the severity of the disease [26]. After 12 h of LPS-induced ALI mice, the expression of COX-2 and iNOS in lung tissue of LPS group was significantly higher than that of vehicle group (Figure 6B,C, *P*<0.01). While after DCXC (10, 20 and 30 mg/kg) intervention, the expression of COX-2 and iNOS in lung tissue of ALI mice was significantly lower than that of LPS group (Figure 6B,C, *P*<0.05, *P*<0.01). These results suggest that DCXC can reduce the protein expression of phosphor NF-κB p65, COX-2 and iNOS in lung tissue of LPS-induced ALI mice.

### DCXC attenuates the binding ability of NF-κB p65 DNA in lung tissue of LPS-induced ALI mice

**Figure 7:**
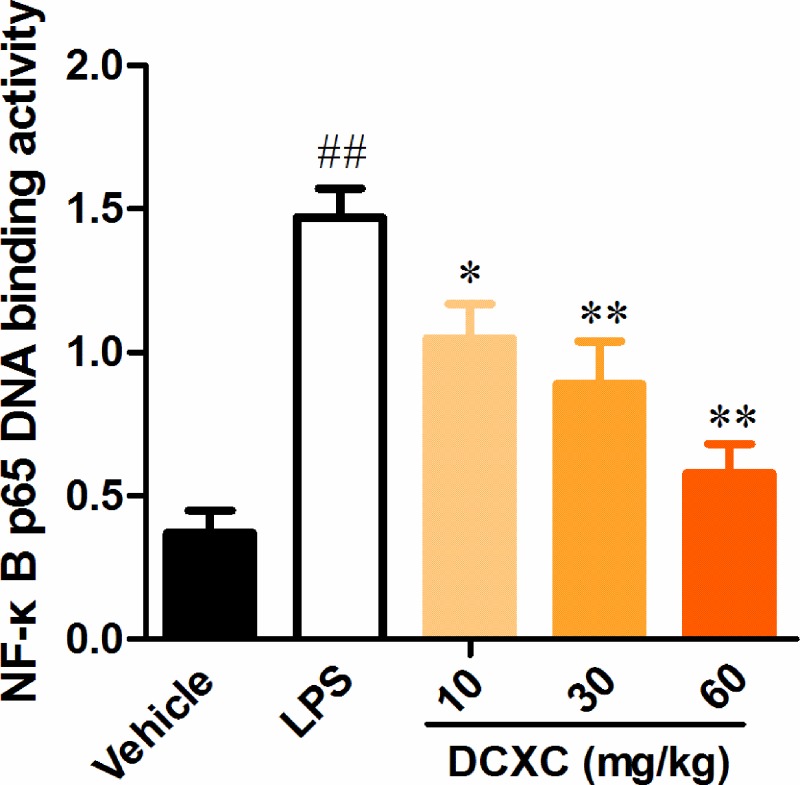
DCXC attenuates the DNA binding ability of NF-κB p65 in lung tissue of LPS-induced acute lung injury mice (*n*=12) ^##^*P*<0.01 vs vehicle group, *P<0.05, **P<0.01 vs LPS group.

After 12 h of LPS-induced ALI mice, the binding capacity of NF-κB p65 DNA in lung tissue of LPS group was significantly higher than that of vehicle group (Figure 7, *P*<0.01). While the binding capacity of NF-κB p65 DNA after DCXC (10, 20 and 30 mg/kg) intervention was significantly lower than that of LPS group, the difference has statistically significant (Figure 7, *P*>0.05, *P*>0.01). The results showed that DCXC could decrease the binding ability of NF-κB p65 DNA in lung tissue of LPS-induced ALI mice, which have a dose-dependent manner.

### DCXC inhibits the mRNA expression of COX-2 and iNOS in lung tissue of LPS-induced ALI mice

**Figure 8:**
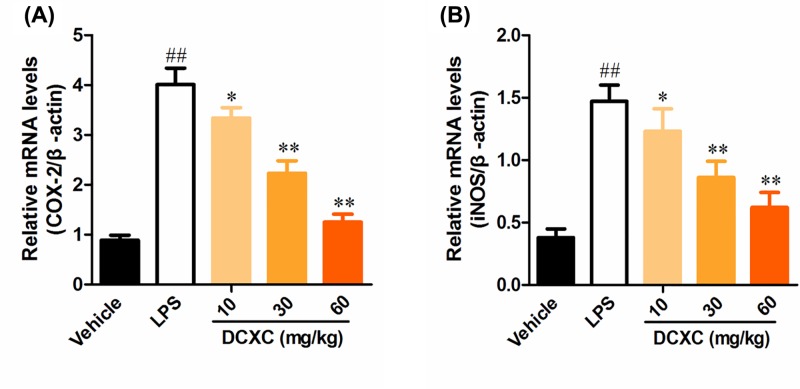
DCXC inhibits the mRNA expression of COX-2 and iNOS in lung tissue of LPS-induced acute lung injury mice (*n*=12) ^##^*P*<0.01 vs vehicle group, *P<0.05, **P<0.01 vs LPS group.

After 12 h of LPS-induced ALI mice, the expression of COX-2 and iNOS in lung tissue of LPS group was significantly higher than that of vehicle group (Figure 8, *P*<0.01). While after DCXC (10, 20 and 30 mg/kg) intervention, the mRNA expression of COX-2 and iNOS in lung tissue of LPS group was significantly lower than that of LPS group (Figure 8, *P*<0.05, *P*<0.01). These results suggest that DCXC can reduce the mRNA expression of COX-2 and iNOS in lung tissue of LPS-induced ALI mice.

## Discussion

Several causes such as sepsis, pneumonia, inhalation of gastric contents, severe trauma, acute pancreatitis and blood transfusion can induce ALI [27]. Among these pathogenic factors, sepsis caused by gram-negative bacteria is the most common pathological state of ALI/ARDS [28]. LPS is the main component of the cell wall of gram-negative bacteria. It can activate macrophages *in vivo* and induce multiple active factors by target cells. LPS can activate TLR4 signal transduction pathway, increase the activity of downstream NF-κB and MAPKs, stimulate the production of TNF-α, IL-1β, IL-6, interferon (IFN), colony stimulating factor (CSF) and other cytokines, and stimulate the production of a large number of reactive oxygen species, causing inflammation [29]. Thus, the LPS components of gram-negative bacteria are considered to play a key role in the inflammatory response of ALI [30]. Therefore, we used LPS to induce ALI model in mice.

The damage of alveolar epithelial cells and vascular endothelial cells are two major pathophysiological changes of ALI caused by different causes. Inflammation and edema are the two most important pathological features [31]. In LPS-induced ALI, neutrophils and macrophages play a major role in inflammatory cells [32]. At the same time, neutrophils and macrophages are also the main sources of various inflammatory mediators *in vivo*, such as TNF-α, IL-6 and IL-1β, which are synthesized and released into tissues and blood [33]. TNF-α is a key inflammatory mediator in LPS-induced toxicity and sepsis. The role of TNF-α in the pathogenesis of endotoxin shock has been extensively studied. Other studies have found that endotoxin shock can occur when recombinant TNF-α is injected into experimental animals [34]. TNF-α may play a role in the formation and progression of endotoxin shock complicated with MOF [35], and TNF-α is an important biomarker in ALI. Studies have shown that TNF-α level in serum and BALF is positively correlated with the severity of shock and mortality of patients. In addition, the increase of IL-1β level was synergistic with the increase of TNF-α level in LPS-induced shock-like state in mice [36]. In the study, it was found that after DCXC treatment with 10, 30 and 60 mg/kg, inflammatory cell exudation and pulmonary edema were alleviated to a certain extent, and the levels of TNF-α, IL-6 and IL-1β in BALF were also reduced to a certain extent in a dose-dependent manner.

MPO, synthesized and expressed mainly by neutrophils, plays an essential role in killing pathogens, especially phagocytosis of bacteria [37]. Neutrophil accumulation is one of the main mechanisms of ALI development, and MPO activity is widely considered as a marker of neutrophil activation and aggregation in inflammatory response [38]. The results showed that the activity of MPO in lung tissue of ALI group increased significantly after LPS stimulation. However, different concentrations of DCXC could decrease the activity of MPO in lung tissue of mice in a dose-dependent manner. Therefore, these results suggest that DCXC intervention can inhibit the accumulation and activity of neutrophils in lung tissue after LPS administration.

NF-κB pathway is an important regulatory pathway of inflammation and immune response in the body [39]. Numerous studies have shown that it plays a key role in ALI [40]. In ALI, LPS also induces the formation of inflammatory mediators through the NF-κB pathway [41,42]. In resting state, IκBα and NF-κB p65, p50 exist in inactivation state. When the upstream signal (TLR4/NF-κB signaling pathway mediated by MyD88) activates IKK (IκB kinase), the activated IKK can ubiquitinate, phosphorylate, and degrade IκBα, which activates the two subunits of NF-κB from inactivation state, transfers them from cytoplasm to nucleus (especially p65 subunit), binds with the corresponding inflammation-related genes, initiates the transcription of inflammatory cytokines, and induces inflammation [43]. Therefore, inhibiting the phosphorylation of IκBα or NF-κB p65 may inhibit the formation of LPS-induced inflammatory mediators to alleviate lung injury and inflammation in ALI [25,44]. In the present study, the results showed that LPS intratracheal injection induced up-regulation of phosphorylation of NF-κB p65 in lung tissue of mice and increased its DNA binding capacity. The ratio of phospho-NF-κB p65/total NF-κB p65 and the binding ability of NF-κB p65 DNA in lung tissue of mice in DCXC group (10, 30 and 60 mg/kg) decreased in a dose-dependent manner, while the levels of phosphor IκBα did not change. The results indicated that DCXC may play a protective role in LPS-induced ALI by inhibiting inflammatory response mediated by NF-κB, and NF-κB p65 may be the action target.

Inflammation is closely related to oxidative stress. NO is not only an inflammatory response but also an important signal molecule in oxidative stress. NO interacts with superoxide free radicals to produce peroxynitrite ions. The iNOS plays a regulatory role in the formation of inflammatory mediators. During inflammation and oxidative stress, iNOS promotes NO synthesis and causes cell damage. The decrease of NO expression was also closely related to anti-inflammatory response [45]. In our study, LPS intratracheal injection can induce up-regulation of iNOS and NO expression in lung tissue of ALI mice. Different doses of DCXC can alleviate the expression of iNOS and NO in different degrees and show a dose-dependent manner.

COX-2 is an important factor downstream of NF-κB signaling pathway in inflammatory response. In ALI model of mice, the expression of COX-2 in lung tissue increased significantly, suggesting that COX-2 also plays an important role in ALI. In addition, inhibiting the expression of COX-2 in lung tissue can alleviate the injury degree of induced lung tissue [46,47]. Our results suggest that LPS induces up-regulation of COX-2 expression in lung tissue of mice, and DCXC may play a protective role in LPS-induced ALI mice by inhibiting COX-2 expression, and DCXC may inhibit COX-2 expression more significantly than iNOS and NO.

As mentioned above, DCXC may play a protective role in LPS-induced lung injury by inhibiting the phosphorylation of NF-κB p65 and the expression of COX-2 and iNOS in lung tissue. However, the target cells of DCXC in this process are still unclear. In LPS-induced ALI model, DCXC can significantly inhibit inflammatory response, which may be related to the inactivation of NF-κB and the down-regulation of COX-2 and iNOS expression. Therefore, DCXC can be used as a potential therapeutic drug for ALI.

In conclusion, DCXC could reduce the number of total cells, neutrophils and macrophages in BALF, reduce the expression of TNF-α, IL-6, IL-1β and NO in BALF, inhibit MPO activity, inhibit the phosphorylation of NF-κB p65, and decrease the expression of COX-2 and iNOS, which suggested that DCXC plays a protective role in LPS-induced ALI in mice.

## Availability of data and materials

The datasets used and/or analyzed during the current study are available from the corresponding author on reasonable request.

## Abbreviations

ALI: acute lung injury
ARDS: acute respiratory distress syndrome
BALF: bronchoalveolar lavage fluid
COX-2: cyclooxygenase
DCXC: Dong Chong Xia Cao
H&E: hematoxylin and eosin
IL-6: interleukin-6
IL-1β: interleukin-1β
iNOS: inducible NO synthase
LPS: lipopolysaccharide
MODS: multiple organ dysfunction syndrome
MOF: multiple organ failure
MPO: myeloperoxidase
NF-κB p65: nuclear factor-κB p65
NO: nitric oxide
PAM: pulmonary macrophages
pNF-κB p65: phosphorylated nuclear factor-κB p65
TCM: traditional Chinese medicines
TNF-α: tumor necrosis factor-α
W/D ratio: wet/dry weight ratio
